# Intensive Care Fundamentals in Romania. A Critical Step in Romanian Intensive Care Education

**DOI:** 10.2478/jccm-2024-0029

**Published:** 2024-07-31

**Authors:** Cosmin Balan, Serban-Ion Bubenek-Turconi, Mo Al-Haddad

**Affiliations:** Institutul de Urgenta pentru Boli Cardiovasculare Prof Dr C C Iliescu, Bucharest, Romania; Carol Davila University of Medicine and Pharmacy, Bucharest, Romania; Queen Elizabeth University Hospital, University of Glasgow, Glasgow, UK

**Keywords:** education, simulation, intensive care medicine

Any doctor working in the field of Intensive Care Medicine (ICM) remembers their first placement in the Intensive Care Unit (ICU). Most would remember that time as being characterized by trepidation, confusion and worry. In order to mitigate these negative experiences, various institutions run ad hoc induction and orientation programs. The European Society of Intensive Care Medicine (ESICM) has recently developed the Intensive Care Fundamentals (ICF) course to standardize induction and orientation to ICU for this group of doctors throughout Europe and beyond. Now, the ICF has arrived in Romania.

The ICF course was developed in 2023 and marks a significant advancement in training and preparation of doctors embarking on an ICU placement. The course’s intended learning outcomes (ILOs) were developed using a modified Delphi technique with the ICU resident in their first month of placement in mind[1]. Employing a hybrid educational model, the theoretical aspects of the ILOs are covered both in the “Intensive Care Fundamentals” book[2] and the eLearning material developed on the eLearning platform of the ESICM, the ESICM Academy (https://academy.esicm.org/). After self-directed learning of the theory, candidates attend a two-day face-to-face course that is characterized by small-group learning, interactive case discussions, interactive workshops and simulation.

Although the ICF book, learning material and teaching slides are written in English, there is a strong emphasis on delivery of the course in the local language and orientation of the candidates to local practices and policies. The course is an opportunity to demonstrate to candidates the applicability of the ICM evidence-base to the local setting.

Romania recently celebrated the launch of the inaugural ICF course in Bucharest, an event that signifies a pivotal development in the nation’s healthcare education provision. Organized by the Centre of Simulation in Anaesthesia and ICM Bucharest (CESIMAB) and hosted at the Centre of Innovation and e-Health of the University of Medicine and Pharmacy Carol Davila Bucharest (CIEH-UMFCD), this initiative was conducted under the auspices of the Romanian Society of Anaesthesia and Intensive Care Medicine (SRATI) (https://www.srati.ro/). This collaboration demonstrates a commitment to enhancing the standard of ICM education and training in Romania, leveraging the country’s established foundation in simulation-based training.

Romania’s engagement with simulation in medical training is not a recent development. The foundation for simulation-based education was laid down much earlier, with a strong emphasis on expanding this capacity recognized by SRATI since the early 2010s. This focus led to the implementation of the “SIMLAB” project, which by 2020 had successfully established four additional simulation centers in Bucharest, Timisoara, Iasi, and Cluj-Napoca, complementing the pioneering center at The George Emil Palade University of Medicine, Pharmacy, Science, and Technology of Targu Mures. These centers were equipped to handle both basic and complex clinical scenarios, enhancing the practical skills of healthcare professionals through high-fidelity simulations.

Moreover, Romania’s commitment to simulation in medical education extends to European collaborations such as the SAFETY project (https://safetymedsim.eu/). Short for Simulation Approach For Education and Training in emergencY, this three-year initiative is part of the EU Erasmus+ program’s Key Action 2. Coordinated by the Department of Medical and Surgical Sciences at the University of Foggia in Italy, the SAFETY project aims to bridge the gap between theoretical knowledge and practical application in Emergency Medicine and simulation. This project, involving Romanian educators from CESIMAB, is enhancing the readiness of medical students and healthcare professionals to handle emergency scenarios efficiently through training using advanced simulation techniques.

The curriculum for the ICF course included case-based scenarios, interactive workshops, and simulation scenarios that mirror real-life clinical challenges that new residents might face. The program was meticulously designed to align with both Romanian medical standards and international quality benchmarks, ensuring that practitioners are well-prepared for the complexities of managing critically ill patients in ICU. Reflecting the ICF philosophy, the program featured small group teaching with 14 trainees divided into two groups, rotating between a total of 7 trainers. Each trainer conducted dedicated sessions, providing focused and personalized instruction to enhance learning outcomes.

The primary goal of the ICF course is to equip new residents with the knowledge and skills necessary for safe and effective practice in ICU settings. The ILOs are extensive, addressing the identification and management of deteriorating patients, execution of daily reassessment routines, specific organ support techniques, and critical non-technical skills such as crisis resource management and ethical considerations in ICM.

The introduction of the ICF course in Romania is more than a local milestone; it is a continuation of a long tradition of excellence in simulation-based medical training, representing a transformative step towards standardizing and enhancing ICM training across Europe. By launching this program, CESIMAB and SRATI have not only set a precedent for other Romanian simulation centres and departments but have also inspired broader improvements in medical education and training nationwide. This initiative is paving the way for training Romanian medical professionals to deliver high-quality care in any intensive care environment.

The feedback on the ICF course was positive. Trainees particularly appreciated the interactive nature of the course and the way complex physiological concepts were elucidated using advanced simulators. Simulation emerged as an ideal method for imparting challenging concepts of applied physiology, such as hemodynamics, acid-base balance, renal replacement therapy, and mechanical ventilation. The hands-on experience and realistic scenarios provided a deeper understanding and retention of these critical concepts, enhancing both the effectiveness and enjoyment of the learning process. Future plans involve the continued application of these concepts at the bedside, ensuring the skills and knowledge acquired are effectively translated into patient care.

Considering the recent adoption of simulation training in the country, having a large proportion of the faculty as young trainers was particularly advantageous. Their familiarity with the latest research and best practices, combined with a unique energy and enthusiasm, fostered a dynamic and progressive learning environment. Additionally, their relatability to young residents enhanced engagement and facilitated effective learning. This generational collaboration could herald the beginning of sustained excellence and innovation in Romania’s healthcare training programs, ensuring the country remains at the forefront of medical education and patient care.

In conclusion, the successful implementation of the ICF course in Romania signifies substantial progress in the country’s medical education provision. It showcases a dedicated effort to embrace and adapt best practices in healthcare education, tailored to meet both global standards and local needs. As Romania continues to develop its healthcare education infrastructure, initiatives like the ESICM’s ICF course will be instrumental in shaping a future where its medical professionals are equipped to provide exceptional and culturally attuned care in any setting.
